# A command centre implementation before and during the COVID-19 pandemic in a community hospital

**DOI:** 10.1186/s12911-023-02394-y

**Published:** 2024-01-08

**Authors:** Liza Grosman-Rimon, Pete Wegier, Ruben Rodriguez, Jane Casey, Susan Tory, Jhanvi Solanki, Barbara E. Collins

**Affiliations:** 1 Humber River Health, M3M 0B2 235 Wilson Ave, North York, Toronto, ON Canada; 2 Institute of Health Policy, Management and Evaluation, University of Toronto, University of Toronto, Toronto, Canada; 3 Department of Family & Community Medicine, University of Toronto, Toronto, Canada; 4 Tampa General Hospital, Tampa, FL USA

**Keywords:** Command Centre, COVID-19 Pandemic, Community Hospital, In-Hospital Acquired Events, In-Hospital Mortality

## Abstract

**Introduction:**

The objective of the study was to assess the effects of high-reliability system by implementing a command centre (CC) on clinical outcomes in a community hospital before and during COVID-19 pandemic from the year 2016 to 2021.

**Methods:**

A descriptive, retrospective study was conducted at an acute care community hospital. The administrative data included monthly average admissions, intensive care unit (ICU) admissions, average length of stay, total ICU length of stay, and in-hospital mortality. In-hospital acquired events were recorded and defined as one of the following: cardiac arrest, cerebral infarction, respiratory arrest, or sepsis after hospital admissions. A subgroup statistical analysis of patients with in-hospital acquired events was performed. In addition, a subgroup statistical analysis was performed for the department of medicine.

**Results:**

The rates of in-hospital acquired events and in-hospital mortality among all admitted patients did not change significantly throughout the years 2016 to 2021. In the subgroup of patients with in-hospital acquired events, the in-hospital mortality rate also did not change during the years of the study, despite the increase in the ICU admissions during the COVID-19 pandemic.Although the in-hospital mortality rate did not increase for all admitted patients, the in-hospital mortality rate increased in the department of medicine.

**Conclusion:**

Implementation of CC and centralized management systems has the potential to improve quality of care by supporting early identification and real-time management of patients at risk of harm and clinical deterioration, including COVID-19 patients.

## Introduction

Healthcare organizations around the world continuously strive to optimize safety and clinical outcomes [1]. In recent years, a few hospitals have taken steps to achieve this by establishing command centres (CC) and employing centralized management systems, which are supported by information technology and real-time data [2–5]. Studies employing a CC have provided evidence for the improvement of operations, patient flow, and clinical decision-making [2, 4, 5]. However, reports on patient outcomes are scarce with one study reporting a decrease in harm scores [3].

Humber River Hospital (HRH) established a CC, employing a high-reliability system, to achieve consistent, predictable, safe, and effective operations. In 2017, 1st generation command centre (CC1) tiles were implemented to improve patient access and flow. In 2019, clinical analytic applications and the world’s first 2nd generation command centre (CC2) tiles were implemented to support early identification and real-time management of patients at risk of harm and clinical deterioration [3]. Unfortunately, in March 2020, the WHO declared the novel coronavirus (COVID-19) outbreak a global pandemic. The effects of incorporating a high-reliability system by implementing a CC in a hospital on clinical outcomes before and after COVID-19 pandemic is not well understood. During the pandemic, hospitals reported that the incidence of in-hospital acquired events such as cardiac arrest increased markedly [6–8], and the rate of survival of these patients was much lower compared to pre-pandemic [7, 8]. In addition, cardiac arrest [9, 10] cerebral infarction [11, 12] respiratory failure [13] and sepsis were also reported to be higher in COVID-19 patients, and their risk of mortality increases as well [7, 9, 10, 14–19].

The objective of the study was to assess the effects of high-reliability system by implementing a CC on clinical outcomes in a community hospital before and during COVID-19 pandemic from the year 2016 to 2021.

## Methods

### Study design

A descriptive, retrospective study was conducted at HRH, Toronto, Ontario Canada. HRH is a fully digital hospital and one of Canada’s largest acute care community hospitals, serving a diverse population of more than 850,000 people in the northwest Greater Toronto Area. HRH operates 722 acute inpatient beds with 3,400 staff, and 700 physicians. HRH is located within the most culturally diverse region in Toronto.

The Humber River Hospital’s CC is a centralized hub where hospital staff can monitor and coordinate all aspects of patient care and hospital operations in real-time. The CC is equipped with advanced technology, including a large video wall, displaying real-time data on patient flow, bed capacity, risk of harm indicators, and other critical information. This real time data enables hospital staff to identify and address issues quickly, such as bottlenecks in patient flow or patient clinical deterioration. The real-time CC software was deployed with key design characteristics to support the care teams who need the information quickly, easily and tuned to a specific situation. The software’s single data architecture connects data elements in real-time and supports rapid response.

The CC uses predictive analytics and machine learning algorithms to anticipate and proactively address potential issues before they occur allowing staff to provide early interventions and prevent adverse outcomes.

The CC employs a high-reliability system, namely, consistently delivering the safest, highest-quality care. This system achieves consistent patient outcomes, enhances operational efficiency, and ensures that the hospital is equipped to provide the highest level of care possible, and effective operations. The study was approved by Veritas ethics review board, an independent ethics review board.

The CC1 was designed to optimize access and flow, as well as transfer of patients from the emergency department (ED) to in-patient units to expedite waiting time and admissions. The CC1 tiles display relevant, real-time data from multiple automated systems across the hospital and form an analytical control panel on large-screen monitors. Decisions can be made to maximize patients’ access and flow, input and throughput in the ED, and output to the in-patient units, which allows for continuum and integrated care, reducing ED length of stay. The CC2 was designed to eliminate risks of patient harm, deterioration and never events by using predictive analytic tiles. The CC2 tiles integrate early warning systems with predictive analytics to generate real-time data from several automated systems that identify any changes in patient conditions and provide early warning. Staff are constantly monitoring for unsafe conditions to be resolved before any harm or adverse events occur.

The CC tiles displayed Covid-19 related data, including a Clinical Deterioration Tile, an Infectious Disease tile, and a Patient Manager Tile. The clinical Deterioration tile was implemented to accelerate care escalation for at-risk in-patients using clinical surveillance algorithms. This Early Warning Score is a clinical algorithm that uses vital signs to help front-line care teams identify patients who are potentially deteriorating. The patient’s COVID-19 status (testing for COVID-19, positive for COVID-19) is also displayed. The Infectious Disease Tile was developed in response to the COVID-19 pandemic. This tile displays a visualization of patient load and critical bed capacity associated with caring for COVID-19 patients. In addition, the information includes a two-week history of hospital COVID-19 testing and results, inpatient locations of in-hospital testing, positive or negative results, recovered patients, critical care bed capacity and critical care EVS status. The analytics support the placement of COVID-19 or COVID-19 suspected patients in the right beds, pinpoints opportunities to accelerate bed cleaning, and prompts teams to work proactively when COVID-19 demand is building (e.g. decant the ICU where possible). The CC staff co-location is comprised of patient admissions coordinators, a bed allocation clerk, a patient flow manager, a homecare manager, a support services supervisor, a medical imaging flow technologist, operating room schedulers and a nursing resource team manager. The co-location of collective skill and expertise of the staff responsible for synchronizing patient admissions improve efficiency in the coordination. The shared workspace is a mechanism for direct and immediate communication. In addition, the CC Clinical Expeditor, a senior nurse, staffed 24/7 monitored COVID-19 patients and patients at risk of deterioration throughout the hospital using the CC analytics. The Clinical Expeditor visited the inpatient units as needed and followed up with staff. These analytics were used to ensure vitals were being monitored based on the HRH Early Warning Score standard, and units were not understaffed when multiple patients were at elevated risk of deterioration, and escalation of patients at elevated risk of deterioration was reported to the physician or rapid response team.

### Data and data sources

Administrative hospital-wide data of in-hospital clinical programs were analyzed from the year 2016 to 2021. During the year 2016 to 2021, there were 3 major events in HRH: (1) in 2017, CC1 was implemented to improve patient access and flow; (2) in 2019, the world’s first CC2 was implemented to support early identification and real-time management of patients at risk of harm and clinical deterioration; and (3) in 2020, the World Health Organization (WHO) declared the novel coronavirus (COVID-19) outbreak a global pandemic. The administrative data included: monthly average admissions, intensive care unit (ICU) admissions, average length of stay, total ICU length of stay (all patients combined), and in-hospital mortality. In-hospital acquired events were recorded and defined as one of the following: cardiac arrest, cerebral infarction, respiratory arrest, or sepsis after hospital admissions. A subgroup statistical analysis of patients with in-hospital acquired events was performed. A subgroup statistical analysis was performed for the department of medicine. The resource intensity weight (WIR), an indicator of the total cost to treat an average in-patient was also assessed.

### Description of command centre

The CC uses a visual display of real-time data with the use of high-reliability principles and predictive analytics, providing clinicians with concurrent information to support decision making [20]. The CCs provide a global view, predictive analytics, and clear protocols to proactively manage operations [21]. The data is continuously monitored 24 h a day, seven days a week by CC staff, detecting risk, coordinating complex care activities, and supporting the healthcare teams in real-time with decision making and patient management,. to reduce risk of harm, identify deteriorating patients early, and eliminate never events [3].

The HRH CC is centrally located in a 4,500 sq. ft. space with 20 workstations, 2 meeting rooms, 4 offices and a 33 liquid crystal display (LCD) screen video wall dedicated to displaying the analytics. Staff seating in the CC is arranged based on their required interaction frequency. The analytics are web-based apps and are accessible via desktop or phone for hospital staff. GE HealthCare CC software is a fully commercialized product (GE HealthCare Technologies, Inc. Chicago, Illinois, USA). CC1 implementation allows teams to organize care delivery activities (e.g., patient discharge), eliminate delays in care, and resolve patient flow bottlenecks (e.g., transferring patients from emergency to an inpatient bed). CC2 implementation focused on early detection and prevention of harm for patients at risk of clinical deterioration and sepsis. Part of digital infrastructure of HRH includes automated laboratory services, robotics for sorting and mixing medications, electronic health records, computerized physician order entry, patient bedside computer terminals, and tracking systems for patients undergoing surgery.

Multiple system and modules around the hospitals communicate with each other to integrate data. The CC tiles display relevant real-time data, from 16 artificial intelligent-powered analytic. The data is integrated from 12 information systems and modules on large-screen monitors. HRH data sources of CC analytics are presented in Table 1.

**Table 1:** HRH data sources of CC analytics

Meditech (Medical Information Technology, Inc. MA, USA)	hospital electronic medical record for patient demographics, location, and clinical documentation
ASCOM (ASCOM Holding AG, Zurich, Swiss)	Nurse to patient assignment and nurse contact information
TDSS (Technology Driven Service Solutions CA)	Portering and housekeeping status
Steris (Steris Mentor, OH USA)	Real-time location systems for operating room flow and patient status
HRH Data Repository	Hospital data lake for operating room schedules, algorithms and more

Information is sent from the source systems using HL7 interfaces and ingested into a central data model. This information is displayed on the CC analytics within 30 s of clinical entry. Some of the data include: (1) clinical deterioration (Identifying in-patients at risk of deterioration and reason for deterioration, and highlighting their monitoring and escalation status); (2) identifying delays in care activities and highlighting deviations from established clinical pathways and best practices; (3) bed state throughout the hospital, surfacing information currently scattered throughout different clinical systems; (4) patients waiting for an inpatient bed in the emergency department; post-anesthesia care unit, birthing unit and clinics outside the hospital; (5) census forecast, providing visibility of current and forecasted inpatient capacity over 48 h time horizon; (6) identifying flow and delays in care activities for the emergency department; and (7) COVID testing activity, critical capacity availability and COVID inpatient population.

Predictive Algorithms used in the CC analytics include: HRH Early Warning Score (vital signs-based algorithm for risk of deterioration); hospital one-year mortality risk (a risk score for patient mortality within one year) systemic Inflammatory Response Syndrome (an algorithm for sepsis risk). Machine Learning algorithms used in the CC include: A census to forecast inpatient capacity over the next 48 h, environmental services forecast to predict housekeeping demand over the next 48 h, and portering forecast to predict portering demand over the next 48 h. Employing CC algorithms has been shown to improve operation and healthcare delivery in several recent studies [2–5].

### Data analysis

Data analysis was performed using IBM SPSS statistics (IBM SPSS Statistics for Windows, Version 23.0. Armonk, NY: IBM Corp). Descriptive statistics were performed for each variable, for each year from 2016 to 2021. Data are presented as monthly average and standard error (SE) or percentage. The analyses of variance (ANOVA) were performed with the Scheffe Post-Hoc tests to compare the difference of the study outcomes between the years. The Kruskal–Wallis test was performed to compare the WIR differences between the years followed by Dunn’s test. The rate of in-hospital mortality was calculated by dividing cases by overall admissions and then multiplying the value by 100. The rates of ICU admissions and mortality of patients with in-hospital acquired events were calculated by dividing cases by overall admissions and multiplying by 100. In the subgroup analysis, the rates of ICU admissions and mortality of patients with in-hospital acquired events were calculated by dividing cases by overall cases of the department of medicine and then multiplying the value by 100.

An independent t-test was used to assess the average monthly differences between 2020 and 2021 in admissions, number of ICU admissions, length of stay, and number of in-hospital mortality cases. A *p*-value of < 0.05 was considered significant.

## Results

Between the years 2016 to 2021, a total of 168,029 patients were admitted to the in-hospital clinical programs of HRH. Among these patients, 11,664 were treated in the ICU, and the total length of stay in the ICU was 92,854 days. The total number of in-hospital mortality cases was 7,548. In these years, 810 patients had in-hospital acquired events (0.48%). Among these patients, 531 were treated in the ICU. The total length of stay was 5,557 days. The number of in-hospital mortality cases was 659 (0.39%). The average age of the patient population for each year from 2016 to 2021 was 49.3, 49.2, 49.3, 48.8, 48.2, 49.0, 49.2 year-of age respectively, and the percentages of male was 40.4%, 40.6%, 40.7%, 40.4%, 40.7%, 40.25 respectively. Health System Performance Measurements of Humber River Health are presented in Table 2.

**Table 2:** HRH health system performance measurements

Hospital Indicators	2016	2017	2018	2019	2020	2021
ED LOS for Admitted Patients (90th Percentile, hours)	21.4	23.9	20.1	20.0	16.4	18.3
ED LOS for Non-Admitted, High Acuity (90th Percentile, hours)	7.3	7.0	7.1	8.0	7.7	8.7
ED LOS for Non-Admitted, Low Acuity (90th Percentile, hours)	4.4	4.1	4.3	5.7	4.8	5.8
Physician Initial Assessment Time (90th Percentile, hours)	3.3	2.9	3.2	4.0	3.1	4.3
Time to Inpatient Beds (90th Percentile, hours)	13.0	16.3	11.8	10.6	6.5	8.0
Ambulance Offload Time(90th Percentile, minutes)	69.0	25.0	23.0	27.0	24.0	35.0

(Definition are found at The Canadian Institute for Health Information CIHI) https://www.cihi.ca/en/access-data-and-reports

The median WIR was not significantly different throughout the years, with a median WIR of 1.15 (IQR 0.58–2.39) in 2016; 1.17 (IQR 0.59–2.42) in 2017; 1.11 (IQR 0.58–2.42) in 2018; 1.05 (IQR 0.53–2.25) in 2019; 1.08. (0.53–2.35) in 2020; and 1.07 (0.54–2.38) in 2021.

### Overall in-hospital clinical outcomes

**Fig. 1:**
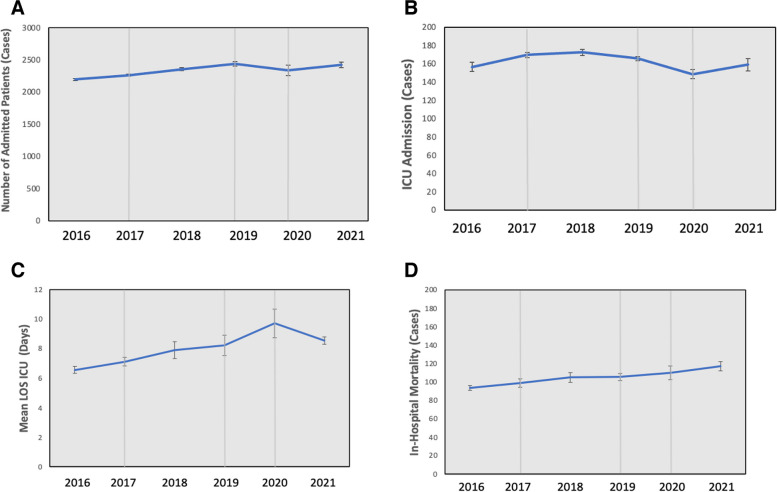
**a** Monthly mean admissions of all in-hospital patients. **b** Monthly mean ICU admissions of all in-hospital patients. **c** Monthly mean length of stay in the ICU (days) of all in-hospital patients. **d** Monthly mean in-hospital mortality of all in-hospital patients. The Y axis represents the average number of cases admitted; X axis represents years. The grey lines represent events: 2017 line represents CC1 was implementation; 2019 line represents CC2; 2020 represents COVID-19 pandemic

The monthly average of hospital admissions increased from 2016, before the implementation of CC, and peaked in 2019 after implementing CC2 (*p* = 0.01), and subsequently dropped at the beginning of the pandemic in 2020 and peaked again in the second year of the pandemic 2021, (*p* = 0.02) (Fig. 1a). There were no changes in the number of ICU admissions, except for a drop in 2020, at the beginning of the pandemic from the number of cases prior to implementing generation 2 CC (*p* = 0.025) (Fig. 1b). The monthly total length of ICU stay was not significantly different during the study years. The monthly mean length of stay increased significantly at the beginning of the pandemic in 2020 from the year prior to CC employment (*p* = 0.016) (Fig. 1c). Overall, in-hospital mortality did not increase significantly throughout the years (Fig. 1d). Similarly, there were no differences throughout the years in the rate of in-hospital mortality to overall admission%.

### Clinical outcomes in COVID-19 patients

In 2020, from March to December, there were 783 COVID-19 cases, with 22.4% (*n* = 176) of the cases treated in the ICU. The total length of ICU stay was 2176 days over this span. The in-hospital mortality among COVID-19 patients in 2020 was 28% (*n* = 220). In 2021, there were 1525 COVID-19 cases, with 26.7% (*n* = 408) of the cases admitted to the ICU. The total length of ICU stay was 4470 days. The in-hospital mortality among COVID-19 patients in 2021 was 16.9% (*n* = 259).

There are no differences in the monthly average of COVID-19 admissions, ICU admissions, total ICU length of stay, average length of stay, and in-hospital mortality between 2020 and 2021 (Table 3).

**Table 3:** Monthly average of COVID-19 patients in 2020 and 2021

Variables	Year 2020 March-December	Year 2021 January-December	*p*- value
**General Admission (cases)**	78.3±18.7	116.9±44.6	0.395
**ICU admission (cases)**	17.6±4.1	31.5±11.6	0.231
**Total LOS ICU (days)**	217.6±48.7	349.4±92.0	0.224
**Average LOS (days)**	17.3±5.3	15.6±2.4	0.724
**in-hospital mortality (cases)**	29.7±6.5	17.6±6.5	0.095

Year 2020: Data are represented as monthly average from March to December 2020

Year 2021: Data are represented as monthly average from January-December 2021

**Fig. 2:**
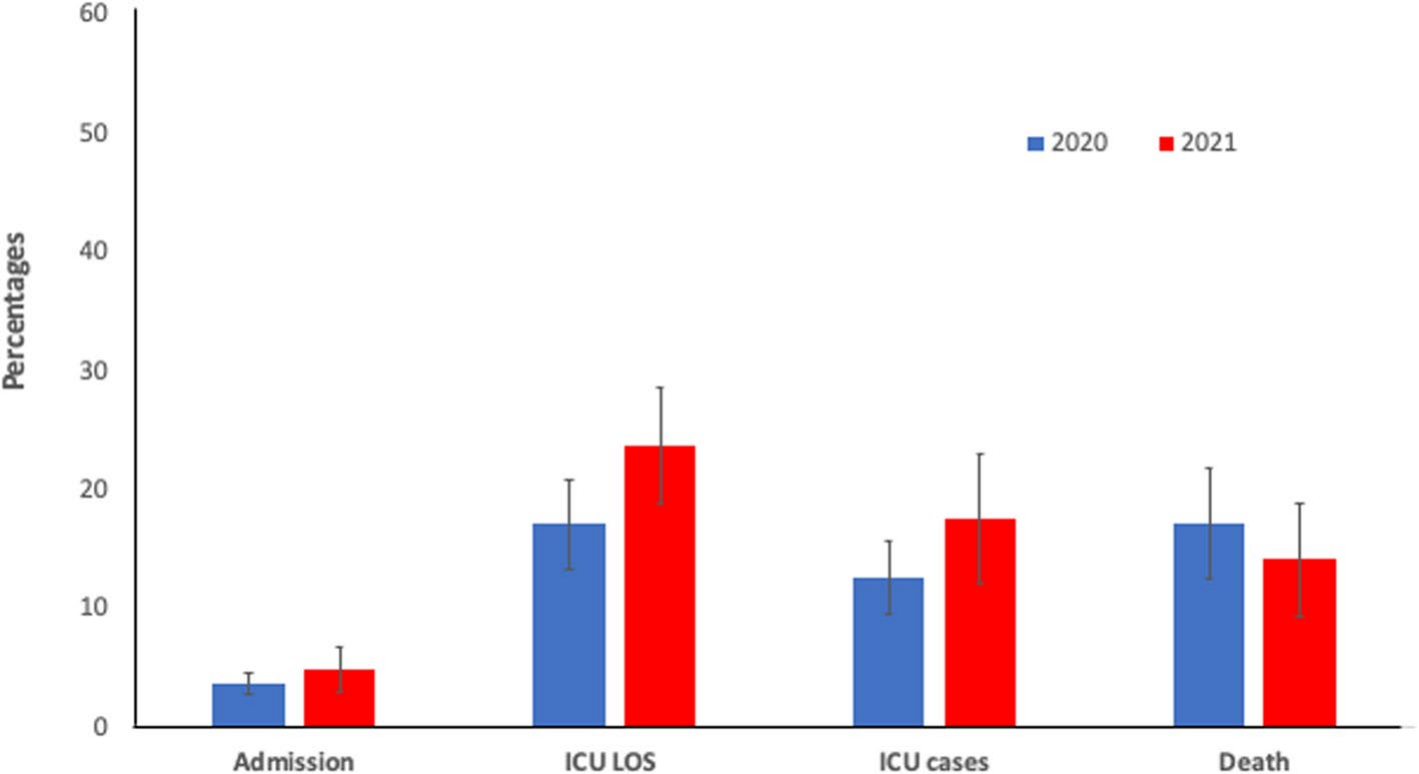
Percentage of COVID-19 Patients from the Entire Patient Population Admitted to the Hospital. The Y axis represents percentages. The X axis represents clinical variables. Blue bars represent the year 2020. Red bars represent the year 2021

 There are no significant differences between 2020 and 2021 in the percentages of COVID-19 from all in-hospital clinical programs in the rate of admissions (3.6±0.9% vs. 4.8±1.8%, *p* = 0.47), ICU admissions (12.6±3.0% vs. 17.5±5.4%, *p* = 0.37), ICU length of stay (17.1±3.7% vs. 23.8± 4.8%, *p* = 0.26), and in-hospital mortality (17.1±4.6% vs. 14.2±4.7%, *p* = 0.45) (Fig. 2).

### Clinical outcomes in patients with In-Hospital acquired events

**Fig. 3:**
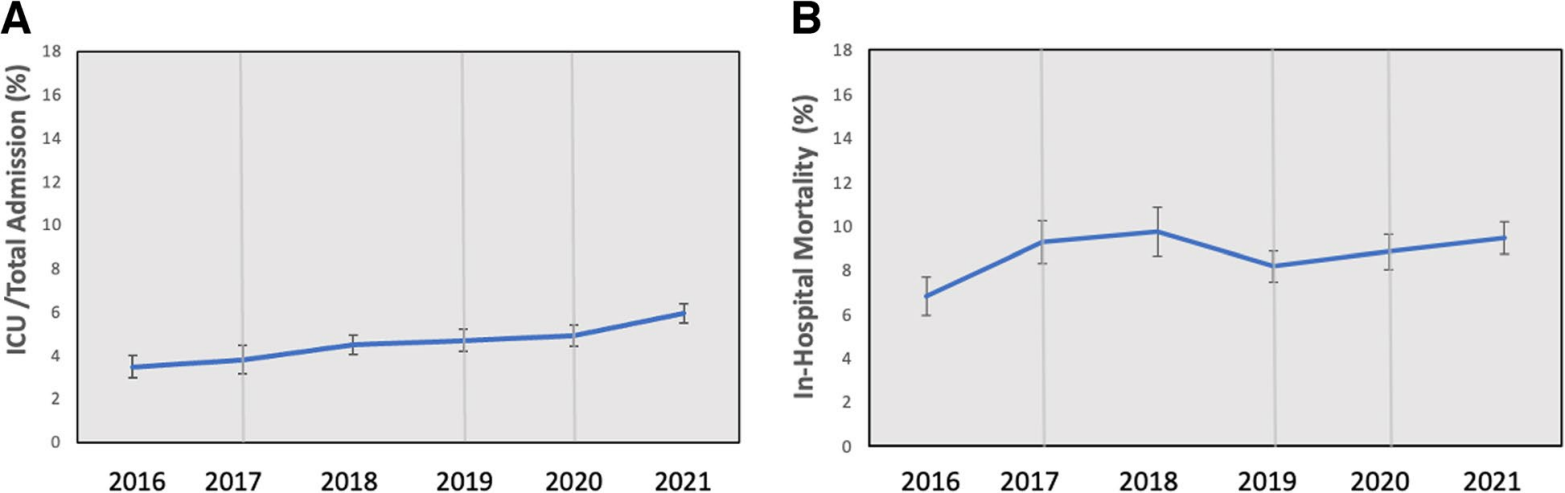
**a** Monthly percentages of ICU admissions/total hospital admissions of patients with in-hospital acquired events. **b** Monthly percentages of in-hospital mortality of patients with in-hospital acquired events / total in hospital mortality cases. The Y axis represents the average number of cases admitted; X axis represents years. The grey lines represent events: 2017 line represents CC1 was implementation; 2019 line represents CC2; 2020 represents COVID-19 pandemic

There were no differences in the rate of in-hospital acquired events to hospital admission% from 2016 to 2021. The rate of ICU admissions of patients with in-hospital acquired events/hospital admission% increased slightly during the second year of the pandemic, in 2021 (*p* = 0.048) (Fig. 3a). There were no differences in the rate of in-hospital mortality of patients with in-hospital acquired events to overall in-hospital mortality cases% (Fig. 3b).

### Clinical outcomes in the Department of Medicine

**Fig. 4:**
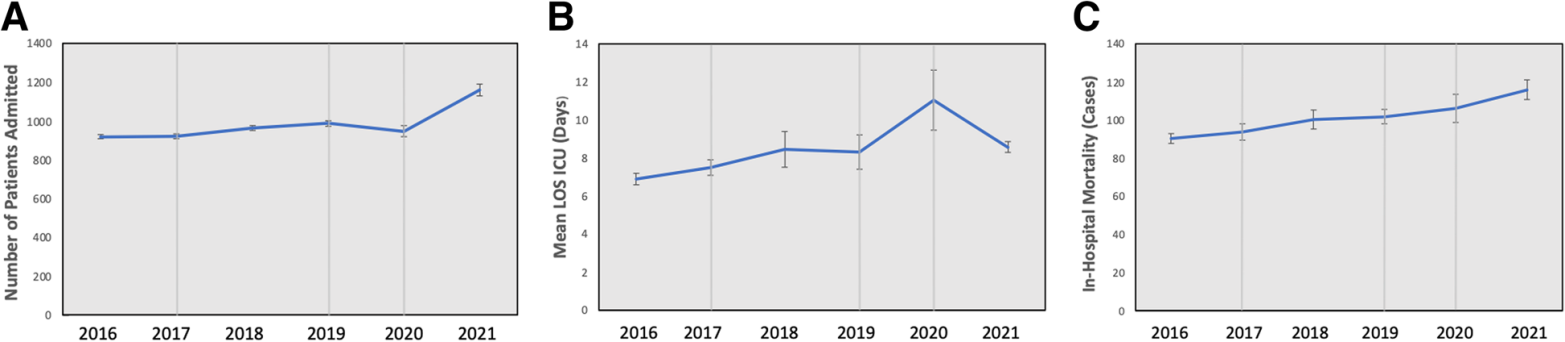
**a** Monthly mean admissions to the Department of Medicine. **b** Monthly mean length of stay in the ICU (days) of patients admitted to the Department of Medicine. **c** Monthly mean in-hospital mortality of patients admitted to the Department of Medicine. The Y axis represents the average number of cases admitted; X axis represents years. The grey lines represent events: 2017 line represents CC1 was implementation; 2019 line represents CC2; 2020 represents COVID-19 pandemic

The number of patients admitted to the department of medicine in 2021 during the COVID-19 pandemic increased significantly from the previous years (*p* < 0.001) (Fig. 4a). However, the number of ICU admissions and the total length of ICU stay of patients admitted to the department of medicine did not change, while the mean length of stays in the ICU increased in 2020 at the beginning of the COVID-19 pandemic (*p* = 0.016) (Fig. 4b). The number of in-hospital mortality cases increased significantly during the COVID-19 pandemic in 2021, compared to all prior years before CC implementation (*P* < 0.001) (Fig. 4c).

In patients with in-hospital acquired events in the department of medicine, the rate of ICU admissions to the overall department of medicine admissions was not significantly different from 2016 to 2021. Similarly, the rate of mortality in patients with in-hospital acquired events to overall morality in the department of medicine was not different.

## Discussion

In this study, we reported data after implantation of a broad hospital-wide CC before and during the COVID-19 pandemic in a community hospital. The major findings of the study were that the rates of in-hospital acquired events and in-hospital mortality among all admitted patients did not change significantly throughout the years 2016 to 2021. While other hospitals around the world observed an increase in in-hospital acquired events and mortality during the pandemic [6–8], we did not observe this pattern. Historical data from Ontario, Canada demonstrated that neighborhoods with a high proportion of immigrants had 4 times higher a risk of COVID-19 infection and 5.2 times higher a risk of death, while neighborhoods with a high proportion of visible minority residents had 3.3 times higher a risk of COVID-19 incidence and 3.5 times higher a risk of death [22]. Although HRH serves the most culturally diverse region in Toronto and is located in the northwest area with the largest concentration of Covid-19 cases [23], we did not observe an increase in mortality rate. Furthermore, despite the increase in ICU admissions in patients with in-hospital acquired events during the COVID-19 pandemic, mortality did not increase. This finding is in line with previous report of our hospital that harm rates was significantly lower in the years 2019–2020 compared to all other hospitals in the province [3].

Furthermore, in the subgroup of patients with in-hospital acquired events, the in-hospital mortality rate also did not change during the years of the study, despite the increase in the ICU admissions during the COVID-19 pandemic. It is important to emphasize that we assessed in-hospital acquired events, a composite of either cardiac arrest, cerebral infarction, respiratory arrest, or sepsis after hospital admissions, while most of the literature reported on one component that we examined – in-hospital cardiac arrest (IHCA). Indeed, our finding was different from previous studies reporting that although hospital admissions, as well as ICU admissions declined during the pandemic, the incidence of IHCA and mortality of these patients was higher [6, 8]. Indeed, patients who had IHCA during the COVID-19 pandemic had overall worse survival compared with those who had an IHCA before the COVID-19 pandemic [7, 8].

In our hospital, there were no significant changes in rates of in-hospital mortality and in-hospital acquired events, including cardiac arrest, cerebral infarction, respiratory arrest, and sepsis before and after the pandemic in our hospital. However, other hospitals around the world observed a surge in IHCA and associated death. A previous study at New York City Health and Hospitals-Jacobi in the Bronx, one of the largest acute hospitals and public health systems in the United States reported that there were 125 IHCAs during a 2.5-month period during the peak of the COVID-19 pandemic, compared with 117 IHCAs in all of 2019 pre-pandemic. A study from Germany reported that hospital admissions declined by 23%, during the pandemic compared to pre-pandemic (13,994 vs. 18,262), but a higher ratio of IHCA to hospital admissions was observed (4.6/1000 vs. 6.6/1000) [6]. A study from Singapore reported that there were more IHCA incidences per 1000 hospital admissions in the COVID-19 period, compared to pre- COVID-19 period (1.86 vs. 1.03), and that the rate of survival to hospital discharge for IHCA was 5.88% in the COVID-19 period as compared to 10.0% in the pre- COVID-19 period [8].

IHCA was significantly more frequent in patients with COVID-19 than in those without, and the severity of illness after IHCA was higher during the pandemic period [6]. A study from Sweden found that COVID-19 was involved in 16% of IHCAs and 10% of all out-of-hospital cardiac arrest (OHCAs), and among COVID-19 cases, 30-day mortality was increased 2.3-fold in IHCA and 3.4-fold in OHCA [10]. Furthermore, COVID-19 has resulted in a low risk of survival, a high rate of hospitalization, and a high number of COVID-19 patients requiring treatment in the ICU [10, 24]. Indeed, in a multicenter cohort study of 68 geographically diverse hospitals across the USA, IHCA was common in critically ill patients with COVID-19 and was associated with poor survival, particularly among older patients and critically ill patients with COVID-19, 14.0% (701/5019) of these patients had in-hospital cardiac arrest, and 57.1% (400/701) of whom received cardiopulmonary resuscitation [9].

The COVID-19 pandemic was associated with a significant reduction in stroke admission rates, but despite that, patients who had strokes during the pandemic had a higher mortality risk [25, 26]. In addition, although infrequent, COVID-19 has been shown to increase the risk of acute ischemic stroke in some patients [11, 12]. Acute ischemic stroke often occurs in COVID-19 patients in the presence of other cardiovascular risk factors, such as hypertension, diabetes, hyperlipidemia, atrial fibrillation, and congestive heart failure [12]. Indeed, ischemic strokes associated with COVID-19 are more severe and more likely to result in severe disability or death [17, 22]. During the pandemic, a greater percentage of COVID-19-positive patients with stroke required treatment in the intensive care units, had longer length of stay, and suffered in-hospital death Compared with COVID-19-negative patients [16]. These reports suggest that stroke in COVID-19 patients increases their risk of mortality. However, overall, we did not observe an increase in mortality rates among all admitted patients.

Moreover, one of the most concerning complications in COVID-19 patients is acute hypoxaemic respiratory failure [13]. COVID-19 patients can exhibit lung damage with low oxygenation index, often leading to acute hypoxic respiratory failure, with small percentages of patients have higher risk of in-hospital mortality [14, 15]. In addition, sepsis also occurred in hospitalized patients with severe cases of COVID-19 [18, 27]. In patients with COVID-19, sepsis is associated with a high in-hospital mortality [19, 28]. In our hospital, overall rates of in-hospital acquired events remained stable before and after the COVID-19 pandemic.

We also observed that although the in-hospital mortality rate did not increase for all admitted patients, the in-hospital mortality rate increased in the department of medicine, suggesting that there is a shift in where in-hospital mortality occurred during the pandemic. This was similar to the previous observation that IHCAs during the COVID-19 pandemic occurred more often in general medicine wards than in ICU [7]. In addition, although the number of patients admitted to the department of medicine increased, in the sub-group of patients with in-hospital acquired events, in-hospital mortality and ICU admissions did not significantly change before and during the COVID-19 pandemic. This was in contrast to a previous study reporting that patients with IHCAs during the COVID-19 pandemic had overall worse survival rates (3% vs. 13%) compared with IHCAs before the COVID-19 pandemic [7].

In our study, WIR was not significantly different throughout the years. Patients with a higher WIR were likely to be more severe cases with a higher mortality rate. The WIR was reported to be positively moderately correlate with mortality in large community hospitals [29]. This suggests that total cost to treat an in-patient was not significantly different through 2016 to 2021.

### Limitations

The study was an exploratory, observational study using administrative data. Whether the lack of change in the rates of in-hospital acquired events and in-hospital mortality among all admitted patients throughout the years 2016 to 2021 was due to the CC should be examined in future studies in a controlled environment. Factors such as vaccination, testing rate, dominant COVID-19 strain severity, and geographical location may affect clinical outcomes associated with COVID-19. Another limitation is that variation in the volume of COVID patients may affect in-hospital acquired events and mortality rate. Indeed, neighbourhoods with the lowest income, visible minority residents, or a higher proportion of immigrants had higher incidences of COVID-19 [22, 23, 30] and increased mortality rates than those with the highest income or with the least immigrants [22, 30]. It is important to emphasize that HRH serves the geographical area of the northwest of the city of Toronto, which had the highest COVID-19 case rates. In the city of Toronto, 53.7% of cumulative cases were diagnosed in 25% of the population, with the largest concentration of cases in the northwest part of the city [23]. Nevertheless, despite the large volume of COVID-19 patients admitted to HRH, our study demonstrated that in-hospital acquired events and mortality did not change significantly before and during the COVID-19 pandemic. In addition, we did not concurrently compare our results to a different hospital, but nevertheless relied on published data from our province, indicating that mortality increase in culturally diverse region in Toronto while in our hospital we did not observe such an increase in our hospital.

As with other technological solutions, CC may improve healthcare delivery, but it is important to examine the effect of design and the implementation of technology. A successful design will improve health care system performance, whereas poor design will impede delivery, increase bureaucracy and costs. Unfortunately, staff and patient surveys were not conducted. Understanding the effects of CC implementation on staff and patients is important to improve process and technological features. In addition, the choice of data and data management are also critical factors for decision making and their effects on healthcare delivery should be also examined.

Process and outcomes measures that are related to response-time to the deteriorating patient, delay in medication administration times and other important variables were not assess and should be investigated in future studies. In our study, we did not examine safety metrics. A study that examined patient safety outcomes, concluded that a CC may improve safety, but it appears that the effect is mainly due to processes around the CC rather than the technological aspects [31]. Further research should evaluate the impact of CC on a variety of patient safety metrics.

We used administrative data of in-hospital acquired events, which include either cardiac arrest, cerebral infarction, respiratory arrest, or sepsis after hospital admissions. It is important that future studies will assess each event separately, since CC implantation may have different magnitudes of effect on cardiac arrest, cerebral infarction, respiratory arrest, or sepsis. An additional limitation of this study is that many confounding variables may affect our observation, including in-hospital acquired events, which consisted of cardiac arrest, cerebral infarction, respiratory arrest, or sepsis. Future studies should confirm our findings and whether the CCs have an effect on different health care quality domains in a controlled environment.

## Conclusion

While other hospitals around the world observed an increase in mortality and in-hospital acquired events (type-2) during the pandemic, we did not observe this pattern. These findings may be explained by the employment of high-reliability systems such as CC.

HRH is the only hospital in Canada that employed CC, and centralized management systems tiles CC2 to improve quality of care by supporting early identification and real-time management of patients at risk of harm and clinical deterioration, including COVID-19 patients. It is possible that our observation would be even more promising oin a post-pandemic area. The preliminary report of CC concept presents a unique digital technological solution that requires rigorous clinical investigation in future studies.

## Data Availability

The data are available upon request from the corresponding author.

## References

[1] Carroll JS, Rudolph JW (2006). Design of high reliability organizations in health care. Qual Saf Health Care.

[2] Chan C, Scheulen J (2017). Administrators leverage predictive analytics to Manage Capacity, streamline decision-making. ED Management: The Monthly Update on Emergency Department Management.

[3] Collins BE (2022). Reducing Hospital Harm: establishing a Command Centre to Foster situational awareness. Healthc Q (Toronto Ont).

[4] Davenport PB, Carter KF, Echternach JM, Tuck CR (2018). Integrating high-reliability principles to Transform Access and Throughput by creating a Centralized Operations Center. J Nurs Adm.

[5] Lovett PB, Illg ML, Sweeney BE (2016). A successful model for a Comprehensive Patient Flow Management Center at an Academic Health System. Am J Med Quality: Official J Am Coll Med Qual.

[6] Roedl K, Söffker G, Fischer D (2021). Effects of COVID-19 on in-hospital Cardiac Arrest: incidence, causes, and outcome - a retrospective cohort study. Scand J Trauma Resusc Emerg Med.

[7] Miles JA, Mejia M, Rios S (2020). Characteristics and outcomes of In-Hospital Cardiac Arrest events during the COVID-19 pandemic: a single-center experience from a New York City Public Hospital. Circ Cardiovasc Qual Outcomes.

[8] Lyu T, Khan FA, Sajeed SM (2021). In-hospital Cardiac Arrest incidence and outcomes in the era of COVID-19: an observational study in a Singapore hospital. Int J Emerg Med.

[9] Hayek SS, Brenner SK, Azam TU (2020). In-hospital Cardiac Arrest in critically ill patients with covid-19: multicenter cohort study. BMJ.

[10] Sultanian P, Lundgren P, Strömsöe A (2021). Cardiac Arrest in COVID-19: characteristics and outcomes of in- and out-of-hospital Cardiac Arrest. A report from the Swedish Registry for Cardiopulmonary Resuscitation. Eur Heart J.

[11] Merkler AE, Parikh NS, Mir S (2020). Risk of ischemic Stroke in patients with Coronavirus Disease 2019 (COVID-19) vs patients with Influenza. JAMA Neurol.

[12] Qureshi AI, Baskett WI, Huang W (2021). Acute ischemic Stroke and COVID-19: an analysis of 27 676 patients. Stroke.

[13] Wilcox SR (2020). Management of Respiratory Failure due to covid-19. BMJ.

[14] Czajkowska-Malinowska M, Kania A, Kuca PJ (2020). Treatment of acute Respiratory Failure in the course of COVID-19. Practical hints from the expert panel of the Assembly of Intensive Care and Rehabilitation of the Polish respiratory society. Adv Respiratory Med.

[15] Li X, Ma X (2020). Acute Respiratory Failure in COVID-19: is it typical ARDS?. Crit Care (London England).

[16] Dhamoon MS, Thaler A, Gururangan K (2021). Acute cerebrovascular events with COVID-19 Infection. Stroke.

[17] Perry RJ, Smith CJ, Roffe C (2021). Characteristics and outcomes of COVID-19 associated Stroke: a UK Multicentre case-control study. J Neurol Neurosurg Psychiatry.

[18] Shappell CN, Klompas M, Kanjilal S, Chan C, Rhee C, Prevalence (2022). Clinical characteristics, and outcomes of Sepsis caused by severe Acute Respiratory Syndrome Coronavirus 2 Versus other pathogens in hospitalized patients with COVID-19. Crit care Explorations.

[19] Abumayyaleh M, Nuñez-Gil IJ, El-Battrawy I (2021). Sepsis of patients infected by SARS-CoV-2: real-world experience from the International HOPE-COVID-19-Registry and validation of HOPE Sepsis score. Front Med.

[20] Collins BE (2021). Use of high-reliability principles in the evolution of a Hospital Command Centre. Healthc Q (Toronto Ont).

[21] Kane EM, Scheulen JJ, Püttgen A (2019). Use of systems Engineering to Design a Hospital Command Center. Joint Comm J Qual Patient Saf.

[22] van Ingen T, Brown KA, Buchan SA (2022). Neighbourhood-level socio-demographic characteristics and risk of COVID-19 incidence and mortality in Ontario, Canada: a population-based study. PLoS ONE.

[23] Mishra S, Ma H, Moloney G (2022). Increasing concentration of COVID-19 by socioeconomic determinants and geography in Toronto, Canada: an observational study. Ann Epidemiol.

[24] Grasselli G, Pesenti A, Cecconi M (2020). Critical care utilization for the COVID-19 outbreak in Lombardy, Italy: early experience and Forecast during an emergency response. JAMA.

[25] Romoli M, Eusebi P, Forlivesi S (2021). Stroke network performance during the first COVID-19 pandemic stage: a meta-analysis based on Stroke network models. Int J Stroke: Official J Int Stroke Soc.

[26] Ganesh A, Stang JM, McAlister FA (2022). Changes in ischemic Stroke presentations, management and outcomes during the first year of the COVID-19 pandemic in Alberta: a population study. CMAJ.

[27] Heubner L, Hattenhauer S, Güldner A (2022). Characteristics and outcomes of sepsis patients with and without COVID-19. J Infect Public Health.

[28] da Silva Ramos FJ, de Freitas FGR, Machado FR (2021). Sepsis in patients hospitalized with coronavirus Disease 2019: how often and how severe?. Curr Opin Crit Care.

[29] Fekri O, Manukyan E, Klazinga N (2021). Associations between hospital deaths (HSMR), readmission and length of stay (LOS): a longitudinal assessment of performance results and facility characteristics of teaching and large-sized hospitals in Canada between 2013–2014 and 2017–2018. BMJ open.

[30] O’Neill B, Kalia S, Hum S (2022). Socioeconomic and immigration status and COVID-19 testing in Toronto, Ontario: retrospective cross-sectional study. BMC Public Health.

[31] Mebrahtu TF, McInerney CD, Benn J et al. Effect of a hospital command centre on patient safety: an interrupted time series study. BMJ Health & care Informatics 2023;30(1). 10.1136/bmjhci-2022-100653 PMC9884873 36697032

